# Multifocal primary cutaneous acral CD8^+^ T-cell lymphoproliferative disorder: An atypical presentation

**DOI:** 10.1016/j.jdcr.2026.06.018

**Published:** 2026-06-15

**Authors:** Haily Fritts, Kelly Frasier, Gabriel Eli Morey, Kelsey Ferrell

**Affiliations:** a Idaho College of Osteopathic Medicine, Meridian, Idaho; b Department of Dermatology, Northwell Health, New Hyde Park, New York; c Spek Diagnostics, Garden City, Idaho; d Treasure Valley Dermatology & Skin Cancer Center, Meridian, Idaho

**Keywords:** acral lesions, cd8 t-cell, cutaneous lymphoma, lymphoproliferative disorder

## Introduction

PCACD8+TCLPD is a lymphoproliferative disorder recently classified in the fifth World Health Organization Classification of Skin Tumors.^1^ Previously grouped under CD8+ acral T-cell lymphomas, it is now recognized as a distinct entity based on clinical, histopathologic, and immunophenotypic features. It typically presents as solitary, slow-growing nodules on acral sites (ears, face, hands, and feet) and follows an indolent course with no systemic involvement. Despite its benign nature, it is underdiagnosed, requiring increased awareness to avoid misclassification and overtreatment.

Epidemiologically, PCACD8+TCLPD is exceedingly rare, comprising <1% of cutaneous T-cell lymphomas.^2^^,^^3^ It predominantly affects middle-aged and older males.^4^ Limited case reports highlight the diagnostic challenges, particularly in distinguishing it from aggressive CD8+ lymphomas. Restricted access to dermatopathology services may delay diagnosis, emphasizing the need for increased clinical recognition. Histopathologically, PCACD8+TCLPD exhibits a dense dermal lymphoid infiltrate of CD8+ T-cells, expressing granzyme B, perforin, and TIA-1, but lacking cytologic atypia seen in aggressive variants.^5^ The absence of epidermotropism, systemic symptoms, and rapid progression helps differentiate it from primary cutaneous aggressive epidermotropic CD8+ T-cell lymphoma, which has a poor prognosis and requires intensive therapy.

This report presents an atypical, multifocal PCACD8+TCLPD case, highlighting diagnostic challenges and management complexities. Accurate diagnosis requires a multidisciplinary approach integrating clinical, histopathologic, and molecular studies. Dermatopathologists play a critical role in distinguishing PCACD8+TCLPD from its aggressive mimics, preventing unnecessary systemic treatments.

## Case report

A 48-year-old male with no significant medical history presented with a pink, translucent, pearly papule with arborizing telangiectasia on the left descending helix for 3 months (Fig 1, *A*). The lesion was mildly tender but asymptomatic, with no systemic symptoms. Clinical suspicion included basal cell carcinoma or squamous cell carcinoma, prompting a shave biopsy. Histopathologic evaluation demonstrated an atypical dermal lymphoid infiltrate composed predominantly of small-to-medium-sized lymphocytes, raising concern for a cutaneous lymphoproliferative disorder and prompting further diagnostic evaluation (Fig 1, *B*). The patient subsequently underwent close clinical follow-up.

Two months later, the left helix lesion enlarged to 1.7 × 1.0 cm and developed tenderness with a rubbery consistency. Additional lesions appeared on the left antihelix and right helix, raising concern for multifocal disease. An excisional biopsy of the left descending helix lesion was subsequently performed to obtain adequate tissue for definitive diagnosis. Histopathologic examination revealed a dense dermal lymphoid infiltrate of small-to-medium-sized T-cells (see Fig 2), with strong CD8 positivity, retained CD5 and CD7 expression, and perinuclear dot-like CD68 staining (see Fig 3). T-cell receptor gene rearrangement studies confirmed clonality, consistent with primary cutaneous acral CD8^+^ T-cell lymphoproliferative disorder.

**Fig 1:**
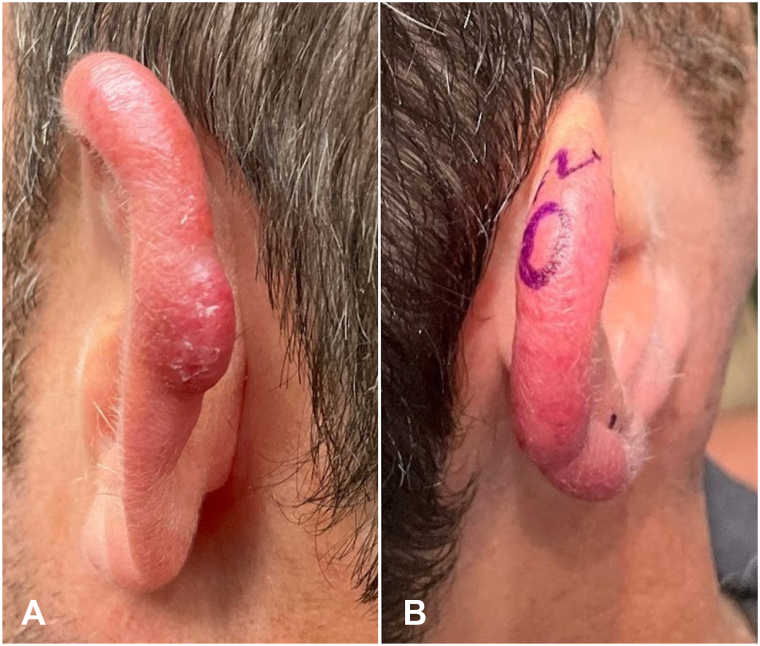
**A,** Initial presentation (7/25): Left ear lesion before biopsy, showing a pink, translucent papule on the descending helix. **B,** Follow-up (9/16): Newly developed right ear lesion, demonstrating similar clinical features.

**Fig 2:**
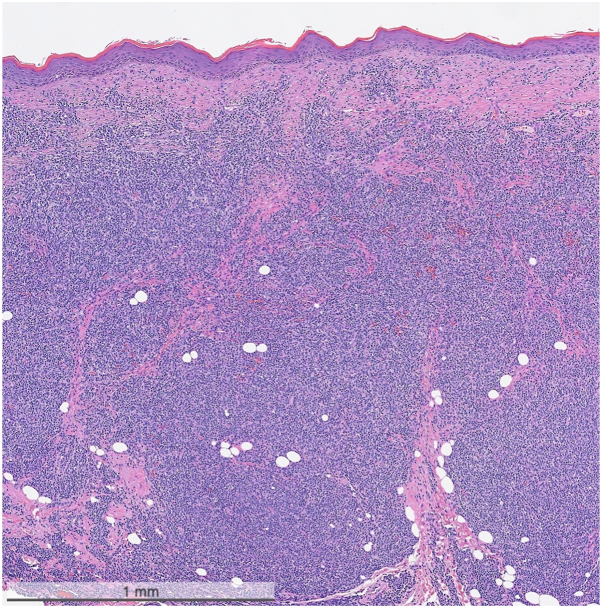
H&E stain (5×) of excisional biopsy specimen, revealing a dense infiltrate of small to medium-sized lymphocytes without distinct architecture, notably lacking exocytosis.

**Fig 3:**
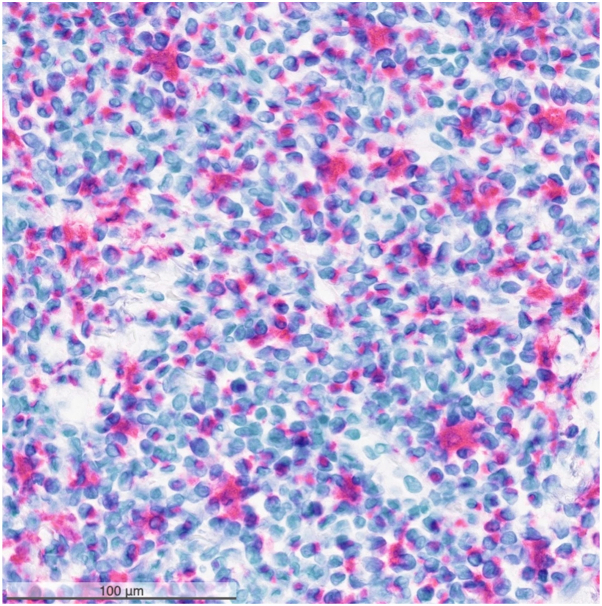
CD68 stain (40×) of excisional biopsy specimen, showing strong perinuclear Golgi staining pattern in most lesional cells, a characteristic feature of PCACD8+TCLPD.

At follow-up, the patient reported a 2-year history of intermittent right helical rim inflammation, worsening over 6 months. Additional biopsies confirmed multifocal PCACD8+TCLPD with atypical features, with partial CD7 loss and an elevated Ki-67 index of 30% to 50% (see Fig 4), raising concerns for peripheral T-cell lymphoma, not otherwise specified. Hematology and oncology evaluation found no systemic involvement, supporting an indolent course. Localized radiation therapy was recommended, with dermatologic surveillance for recurrence or progression. The patient remains under close observation with localized treatment under consideration.

**Fig 4:**
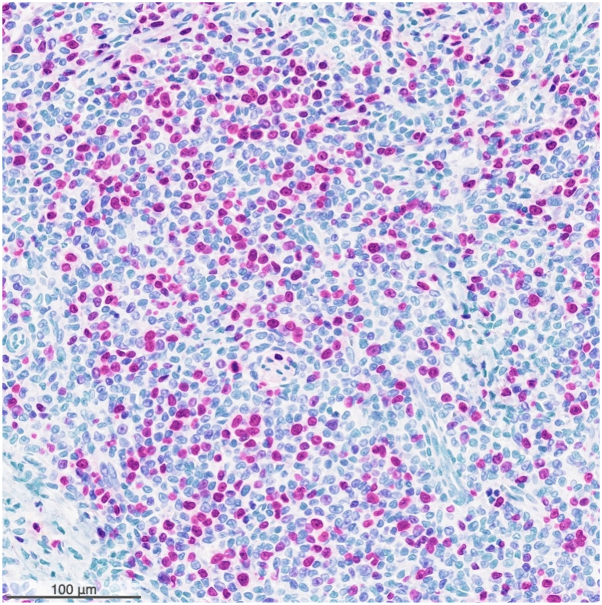
Ki-67 stain (20×) of follow-up biopsy specimen, demonstrating a high proliferative rate (30% to 50%).

## Discussion

### Epidemiology and clinical presentation

PCACD8+TCLPD is a rare, indolent disorder affecting middle-aged and older adults, with a slight male predominance. It typically presents as a solitary, slow-growing nodule on acral sites (ear, face, and feet).^6^^,^^7^ Multifocal cases, as seen in this case, are rare and complicate management. Unlike aggressive CD8+ T-cell lymphomas, PCACD8+TCLPD lacks systemic involvement, angioinvasion, or significant cytologic atypia. KIR3DL1 gene alterations have been implicated in its pathogenesis,^8^ though further research is needed.

While generally indolent, some cases show rapid growth and local tissue destruction.^6^ However, recurrence risk necessitates long-term monitoring. Distinguishing it from other CD8+ lymphomas, particularly those with central nervous system involvement, is critical for appropriate management.^9^

### Immunophenotypic and histological features

Histologically, the lesion is characterized by a monomorphic population of small to medium lymphocytes without exocytosis. The immunophenotypic profile of PCACD8+TCLPD remains a cornerstone of its diagnosis, offering clues to distinguish it from aggressive CD8+ T-cell lymphomas. Immunohistochemical staining typically shows strong CD8 positivity with co-expression of cytotoxic markers such as granzyme B, perforin, and TIA-1.^5^ Perinuclear dot-like CD68 staining, often described as a hallmark of this disorder, reflects the unique cytotoxic granule distribution within the tumor cells.^8^ Epidermal sparing with a well-defined grenz zone further aids differentiation from other T-cell lymphomas that commonly exhibit epidermotropism.^10^ Molecular studies, including T-cell receptor gene rearrangement analysis, are indispensable for confirming clonality, particularly in challenging cases with atypical features. Although Ki-67 proliferation indices in PCACD8+TCLPD are typically low, cases with indices above 30%, as seen in this patient, warrant a more cautious clinical approach, given the potential for localized aggressive behavior.

### Treatment and management

Management of PCACD8+TCLPD is typically conservative given its indolent clinical course. Most reported cases are treated with local surgical excision or localized radiation therapy, both of which demonstrate excellent outcomes in solitary lesions.^11^ In multifocal presentations, treatment decisions may be individualized based on disease burden, symptoms, and cosmetic considerations. Long-term follow-up with periodic clinical dermatologic examinations is recommended, as additional lesions or local recurrence may occur despite the generally favorable prognosis. Systemic staging is typically reserved for patients with atypical clinical features or concern for more aggressive disease.

## Conclusion

This case examines the diagnostic and therapeutic complexities associated with the atypical presentation of multifocal PCACD8+TCLPD involving bilateral ears-a rare manifestation of an already uncommon disorder. These unique clinical and histopathological challenges highlight the importance of accurate diagnosis to ensure appropriate, targeted management and to prevent both overtreatment and undertreatment. Prompt recognition of atypical features, such as multifocality and elevated proliferative indices, requires a multidisciplinary approach, with close collaboration among dermatologists, pathologists, oncologists, and radiation oncologists to deliver individualized care. Effective management depends on diagnostic precision and individualized therapeutic strategies, which directly determine patient quality of life while preventing misdiagnosis-driven overtreatment and unnecessary interventions.

## Conflicts of interest

None disclosed.
